# Current Status of Studying on Physiological Mechanisms of Rice Response to Flooding Stress and Flooding-Resistant Cultivation Regulation

**DOI:** 10.3390/plants14182863

**Published:** 2025-09-14

**Authors:** Weicheng Bu, Irshad Ahmad, Han Fei, Muhi Eldeen Hussien Ibrahim, Yunji Xu, Tianyao Meng, Qingsong Zuo, Tianjie Lei, Guisheng Zhou, Guanglong Zhu

**Affiliations:** 1 Joint International Research Laboratory of Agriculture and Agri-Product Safety, the Ministry of Education of China, Yangzhou University, Yangzhou 225009, China; mz120231391@stu.yzu.edu.cn (W.B.); xuyunji19881004@163.com (Y.X.); tymeng@yzu.edu.cn (T.M.); 2 Jiangsu Provincial Key Laboratory of Crop Genetics and Physiology, Agricultural College, Yangzhou University, Yangzhou 225009, China; irshadgadoon737@yahoo.com (I.A.); gszhou@yzu.edu.cn (G.Z.); 3 Jiangsu Co-Innovation Center for Modern Production Technology of Grain Crops, Agricultural College, Yangzhou University, Yangzhou 225009, China; mz120231410@stu.yzu.edu.cn; 4 Faculty of Agriculture, University of Science and Technology of Sudan, Khartoum 11115, Sudan; mhsinada@yahoo.com (M.E.H.I.); qszuo@yzu.edu.cn (Q.Z.); 5 Institute of Environment and Sustainable Development in Agriculture, Chinese Academy of Agricultural Sciences, Beijing 100081, China; leitianjie@caas.cn

**Keywords:** flooding stress, physiological mechanisms, resource use efficiency, grain yield, cultivation regulation

## Abstract

Due to climate change, flooding stress has occurred more frequently and intensively than ever before, which has become one of the major abiotic stresses affecting rice production. In tropical regions around the world, southeastern coastal countries, and southern rice production areas of China, frequent flooding disaster usually takes place during the rainy season and heavy summer rainfall, which leads to great yield losses in rice production. Currently, only a few rice genotypes are flooding-tolerant, and the relevant flooding-resistant cultivation and regulation practices are still lacking. Therefore, this review highlighted the latest studies on the physiological mechanisms of rice response to flooding stress and flooding-resistant cultivation, particularly summarizing the effect of flooding stress on rice root system architecture, plant growth, reactive oxygen metabolism, energy metabolism, radiation use efficiency, endogenous hormone metabolism, nitrogen utilization efficiency, and yield formation. In addition, the breeding strategies and cultivation regulation approaches for alleviating the flooding stress of rice were analyzed. Finally, future research directions are outlined. This review comprehensively summarizes the rice growth performance and physiological traits response to flooding stress, and sums up some useful regulation strategies, which might assist in further interpreting the mechanisms of plants’ response to flooding stress and developing stress-resistant cultivation practices for rice production.

## 1. Introduction

Rice (*Oryza sativa* L.) is an important food crop worldwide, which faces serious problems from flooding and waterlogging during the growing season [1]. Due to faster global climate change, the frequency and intensity of extreme precipitation events have become more frequent and severe, establishing flooding as a major stress factor limiting rice production [2]. Research showed that in the past decade, global food production cuts have reached an average of 9% annually, and the yield loss caused by continuous flooding in the main rice-producing areas in Southeast Asia and South Asia accounts for more than 60% [3,4]. Due to the influence of the monsoon climate, heavy summer rainfall occurs frequently. Over the past 20 years, extreme waterlogging or flooding events have led to an average reduction in rice yield of approximately 7.6% [5]. Flooding disasters in China have caused significant losses to agricultural production. In 1998, 29 provinces experienced flooding, affecting 22.29 million hectares of land and resulting in 49 million tons of grain loss [6,7]. In 2021, the flooding of early rice in Zhejiang Province during the plum rain season affected 455,600 hectares, leading to an average yield reduction of approximately 6.5% [8]. Flood stress negatively affects plant growth and development, and yield formation of rice through impairing soil aeration, nutrient absorption, and photosynthetic efficiency [9]. Flood disasters have become one of the primary abiotic stress factors limiting agricultural productivity.

In recent years, despite advances in rice breeding and yield potential, flood resistance remains limited in different new varieties [10]. Waterlogging severely affects the crop’s growth, development, quality, and yield. Previous research showed that rice tillering has decreased dramatically under flooding stress and as a result has reduced the yield [11,12]. During the early growth period, the number of rice tillers decreased by 72.2%, and the yield dropped by 65.7% under semi-flooding stress. This sharp yield reduction was mainly attributed to a decrease in resource use efficiency such as light energy and nitrogen fertilizer caused by flooding stress, further confirming the significant inhibitory effects of flooding on rice growth. Currently, relevant research on the effects of flooding stress in rice has primarily focused on its impact on above-ground growth, physiological characteristics, and yield formation [13,14]. In terms of post-flood recovery, existing research primarily focuses on fertilizer management and other practices that support rice regrowth and physiological recovery following flooding and drainage [15]. The impact of flooding stress on the morpho-physiological activity of the rice root system has already been well studied. However, the physiological mechanism of resource utilization efficiency (light energy, nitrogen use efficiency) and yield formation of rice response to flooding stress is still unclear. In addition, waterlogging-resistant cultivation measurements are particularly lacking, especially in terms of preventive measures before flooding and control strategies during flooding.

Based on this, the present review highlights the physiological mechanism of rice in response to flooding stress and recent advances in flooding-resistant cultivation practices. It analyzes the effects of flooding stress on rice root system development, plant growth, reactive oxygen metabolism, energy metabolism, light use efficiency, regulation of endogenous hormones, nitrogen use efficiency, and yield. Additionally, it presents breeding strategies and cultivation management methods to mitigate flood stress in rice, and proposes future research directions. This investigation provides a theoretical foundation for the selection and breeding of flood-resistant rice varieties and the development of high-yield, flooding-resilient cultivation techniques.

## 2. The Concept, Type, and Occurrence Characteristics of Rice Flooding

Rice can generally grow in a 5 to 10 cm water layer (varies depending on the breeding stage of the rice) [16]. When the water accumulation depth exceeds more than 15 cm, it leads to poor growth or even plants’ death due to waterlogging [17]. According to the water level and degree of the flooding, flooding can be divided into three categories: (1) complete flooding; rice plants are completely submerged, often occurring in seed germination, the seedling stage and tillering stage, and the water depth is nearly from 10 to 25 cm; (2) half-flooding; the flooding depth is about half of the plant height, where a water depth of 25 to 50 cm stagnates in the field, often occurring in the seedling stage and tillering stage, and occurring from time to time; and (3) deep flooding; the flooding depth is between or above 50 cm, often occurring in low-lying rice areas and deep rice cropping areas in southeastern coastal countries [18]. In rice production, early-stage semi-flooding frequently occurs during population establishment and can cause substantial damage to rice development.

## 3. Effects of Flooding Stress on Growth, Physiology, and Yield

### 3.1. The Effect of Flooding Stress on Rice Plant Growth Traits

**Figure 1:**
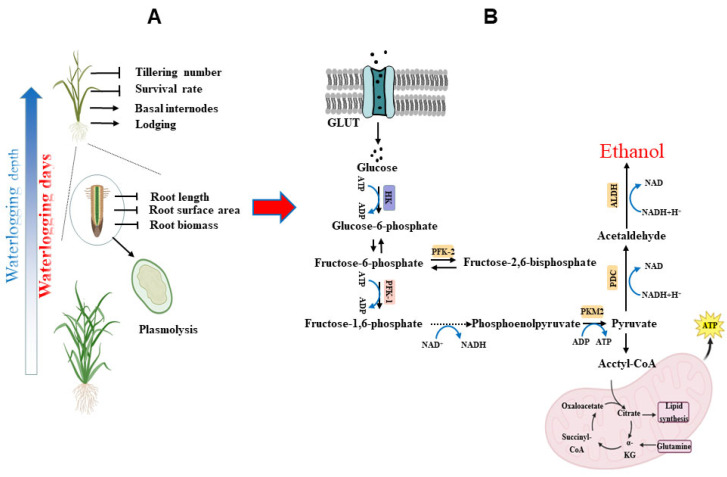
Effects of flooding stress on root architecture (**A**) and physiological metabolism (**B**) of rice. Abbreviations: GLUTs: glucose transporters, HK: hexokinase, PFK: phosphofructokinase, PDC: pyruvate decarboxylase, ALDH: aldehyde dehydrogenase, PKM: pyruvate kinase muscle isoform, ATP: Adenosine Triphosphate.

Flooding stress seriously affects rice growth and development. It alters the dry matter allocation coefficient among the various organs of rice. The dry matter allocation coefficient is highest in the leaves, followed by stems, while it decreases in the roots and shows the greatest reduction in the spikelets. The increase and decrease trends become more significant with the increase in the degree of flooding stress [19]. Rice yields are reduced under different flooding stresses; submerged flooding causes higher yield reductions than partial flooding, and the longer flooding durations lead to more serious yield losses [20]. Previous research showed that the impact of flooding stress on rice varies across different growth stages. Half-flooding stress lasting more than 2–3 days can affect rice growth, inhibit tillering, and thus affect the yield. During the early stages of seed germination and population construction, over 3–5 days of submersion can lead to complete death of the crop (Figure 1) [21]. Semi-flooding damage will occur during key breeding periods such as the seedling stages and panicle differentiation stages, which will have a fatal impact on rice growth and production [22]. Under semi-flooded waterlogging stress, the most notable characteristics of rice growth are a sharp decline in tillering, a significant increase in elongation of the basal internode, a severe reduction in the survival rate, serious lodging after drainage, and substantial yield loss (Figure 1). The above-mentioned manifestations of rice varieties that are not water-resistant are particularly serious, such as Zhuliangyou171, Zhongjiazao17, Xiangzaoxian45, Wuyujing 3, etc. [23,24].

**Figure 2:**
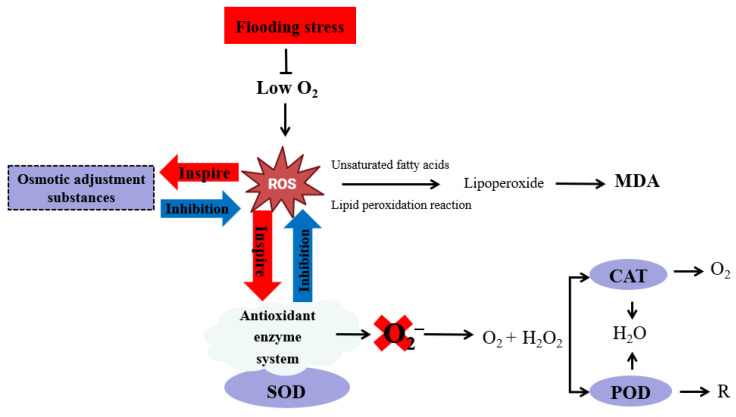
Impact of flooding stress on ROS (reactive oxygen species) metabolism in rice. Flooding stress reduces oxygen uptake in rice, leading to excessive accumulation of reactive oxygen species (ROS: H_2_O_2_, O_2_^−^, ·OH). ROS induce lipid peroxidation reactions, generating MDA as a harmful byproduct. To mitigate oxidative damage, plants employ two primary strategies: (i) activation of the enzymatic antioxidant system: ROS trigger the upregulation of antioxidant enzymes. SOD catalyzes the dismutation of O_2_^−^ into H_2_O_2_ and O_2_. Subsequently, CAT and POD detoxify H_2_O_2_ into harmless H_2_O and O_2_; (ii) activating the non-enzymatic antioxidant system: plants alleviate ROS toxicity through the synthesis of secondary metabolites. Abbreviations: MDA: malondialdehyde, SOD: superoxide dismutase, CAT: catalase, POD: peroxidase. R: harmless oxidation products.

Quiescence and escape are the two major adaption strategies in rice to cope with flooding stress. Under flooding conditions, the survival rate of rice plants significantly drops, and the survival rate mainly depends on the elongation of the plant stems. In a low O_2_ environment, rice is forced to rely on anaerobic respiration to obtain energy, resulting in a reduced ATP production rate that cannot meet the energy demands of growth (Figure 2) [25]. On the other hand, flooding stress induces accelerated ethylene synthesis, which in turn regulates gibberellin production, prompting the plant to rapidly elongate in an effort to obtain oxygen—an adaptive, yet often irreversible, response (Figure 1) [26]. This is named the escape strategy. Especially under semi-flood conditions, flooding-sensitive rice consumes a lot of energy (non-structural carbohydrate, NSC) to accelerate stem elongation, avoiding submersion and thus ensuring survival (Figure 2) [27]. Therefore, stem elongation in flooding-sensitive rice significantly increased under flooding conditions. However, in the quiescence strategy, the extent of elongation is considerably far less in flooding-tolerant varieties compared to sensitive ones [28]. This is mainly because flooding-tolerant varieties maintain higher carbohydrates and an optimal ethanol fermentation rate [29], slowing down or even no stem elongation, and laying a material foundation for rapid growth recovery after flooding. In the escape strategy, most of the energy substance is consumed for stem elongation, resulting in energy deficits for tillering and growth, which may be one of the reasons for the inhibition of and reduction in rice tillering under semi-flooded stress. In previous studies, we also confirmed that the reduction in rice tillering under semi-flood conditions is closely related to stem sheath NSC [12].

### 3.2. The Effects of Flooding Stress on Rice Roots’ Growth Traits

Under flooding stress, the rice root system is the first organ to detect flooding and the response to flooding conditions. The biomass of the rice root system was significantly reduced, which was caused by decreases in the root length, the number of root tips, and root strength (Figure 1A). Due to flooding, the root system becomes thinner and the number of root hairs decreases, leading to a decreased contact area between the roots and the soil, which in turn weakens the plant’s nutrient uptake capacity [30]. The toxic substances produced by the enhanced activity of anaerobic microbes further affect the root system’s nutrient absorption [31]. Flooding stress accelerates the separation of cell walls in parenchymal cells in the root mesocortex, causing the plasmolysis cells to converge and form large cavities, which are named aerenchyma (ventilated tissue) [32]. Aerated tissue transports oxygen from the aerobic site of rice to the hypoxic root system, which decreases the radial O_2_ loss (ROL) in rice roots under flooding conditions and is an adaptation strategy for rice to flood stress [32]. Previous research showed that flooding significantly reduced rice root biomass and length, weakened root toughness and mechanical strength, and led to the formation of extensive aerenchyma (ventilated tissue). The degree to which the ventilated tissue formed in distinct parts of the root system and rice varieties with varying flood resistance abilities differed greatly. In general, the formation of thinner cell walls and aerenchyma, as well as a decrease in the roots’ bleeding intensity in the root, leads to weakened root strength under flooding conditions.

The physiological metabolism of the root system suffered in two stages from aerobic to anaerobic under flooding stress (Figure 1B): (1) Under hypoxic conditions, root cells undergo plasmolysis, resulting in significant reductions in root length, root surface area, and biomass. Plasmolysis of root cells disrupts water and nutrient uptake, which in turn impairs overall plant vigor. This consequence leads to a reduction in the tiller number and survival rate. (2) In the physiological metabolism, glucose molecules enter the cytoplasm via facilitated diffusion or active transport mediated by specific membrane transporters, glucose transporters (GLUTs). Glycolysis subsequently converts glucose to pyruvate, which enters the mitochondria. Then, pyruvate undergoes oxidative decarboxylation to form acetyl-CoA. Acetyl-CoA then enters the citric acid cycle, coupling with oxidative phosphorylation to generate energy. Pyruvate is enzymatically converted to ethanol and carbon dioxide.

### 3.3. Effects of Flooding Stress on Reactive Oxygen Metabolism

Under flooding stress, rice plants produce large amounts of reactive oxygen species (ROS), such as hydroxyl radical (·OH) and superoxide anion (·O_2_^−^), with O_2_^−^ particularly notable for being converted into hydrogen peroxide (H_2_O_2_) and the more harmful ·OH (Figure 2). These ROS trigger membrane lipid peroxidation (resulting in malondialdehyde production) and diacylation, directly damaging nucleic acids and proteins. This leads to structural damage to nucleic acids, protein cross-linking and polymerization, polypeptide chain breakage, enzyme inactivation, and other detrimental effects [33,34]. Previous research showed that under semi-flooded waterlogging conditions, the malondialdehyde (MDA) and H_2_O_2_ content in rice increased by 96.8% and 97.5%, respectively. These oxidative damages lead to promoted chlorophyll decay in leaves and altered enzyme activity, which ultimately lead to limited plant growth [35].

Plants generally relieve or eliminate the harmful effects of ROS through two ways (Figure 2). One mechanism involves the increased activity of antioxidant enzymes such as superoxide dismutase (SOD), peroxidase (POD), and catalase (CAT). SOD converts ·O_2_^−^ into H_2_O_2_ and O_2_. The resulting H_2_O_2_ is then broken down into non-toxic H_2_O and O_2_ by CAT and POD, thereby protecting the plant from oxidative damage [36]. The functions of CAT and POD respond to clear H_2_O_2_ in cells, but their position and mechanism of action in cells are different. CAT directly catalyzes the decomposition of H_2_O_2_, while POD indirectly eliminates H_2_O_2_ by catalyzing ascorbate peroxidases and others. They both play a key role in blocking free radical chain reactions [37]. Another pathway is a non-enzymatic system (or osmoregulatory system), where plants neutralize or alleviate damage to harmful substances by producing osmotic regulators [38]. The non-enzymatic antioxidant system comprises low-molecular-weight compounds that mitigate oxidative damage in cells and tissues through direct neutralization of free radicals and ROS [33]. Primary constituents include ascorbic acid (vitamin C), α-tocopherol (vitamin E), and the tripeptide glutathione (GSH). Under normal circumstances, the production and removal of reactive oxygen species in the plant body are in a dynamic equilibrium state. In adverse stress conditions such as waterlogging, plants produce excessive amounts of harmful substances such as ROS, including ·OH, ·O_2_^−^, and H_2_O_2_. These ROS cause oxidative damage to cellular components, disrupt physiological processes in plants, and, if not effectively managed by antioxidant systems, can lead to irreversible injury or even plant death tissues.

### 3.4. The Effects of Flooding Stress on Radiation Use Efficiency

**Figure 3:**
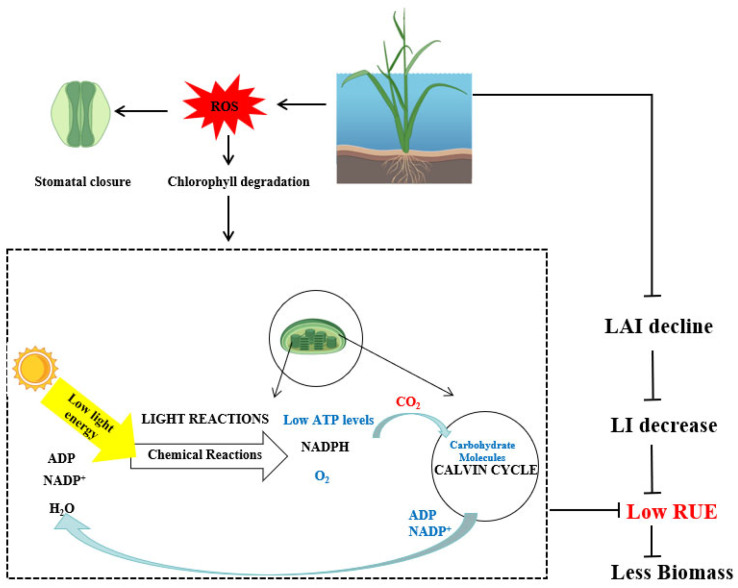
Impact of flooding stress on radiation use efficiency in rice. Flooding submerges rice leaves, reducing the LAI and light interception, thereby decreasing RUE. Concurrently, waterlogging-induced ROS trigger stomatal closure. This restricts the conversion of solar energy into chemical energy by chloroplasts within the leaves, resulting in diminished ATP production and impaired photosynthesis. The consequent reduction in photosynthetic efficiency further lowers RUE, ultimately leading to decreased rice biomass. Abbreviations: LAI: leaf area index, LI: light interception, RUE: radiation use efficiency.

Radiation use efficiency (RUE) refers to the effectiveness with which light energy intercepted by the plant canopy is converted into biomass. It involves two key processes: light interception and light energy conversion (Figure 3). Light interception mainly depends on the leaf area index of the population, while the photoenergy conversion efficiency mainly depends on the photosynthesis of the blades [39]. Approximately 90% of rice yield comes from photosynthesis in leaves [40]; therefore, light energy utilization is a key determinant of both crop biomass and grain yield. When the rice plants are completely submerged, the light intensity, and O_2_ and CO_2_ concentration and transmission rate drop, among which low light and low CO_2_ directly inhibit the photosynthesis of rice and reduce RUE [41]. Previous research demonstrated that flooding-induced reductions in the rice tiller number led to a smaller canopy and lower leaf area index (LAI), which limited the plants in intercepting photosynthetically active radiation (PAR) and significantly reduced RUE (Figure 3). In addition, under flooding conditions, the photosynthetic pigment content of rice leaves significantly decreases, stomatal closure reduces CO_2_ absorption, and the activity of the key enzyme Rubisco is inhibited, collectively impairing photosynthetic efficiency and RUE [42]. The changes in chlorophyll fluorescence parameters can be used as an indicator to evaluate the efficiency of light energy utilization. Chlorophyll fluorescence parameters in rice vary significantly across different growth stages and under varying flood depths [43]. Spectral monitoring technology can be used to evaluate the light energy utilization efficiency of rice under flood stress. By analyzing the canopy spectrum characteristics, a model related to the light energy utilization efficiency can be established, thereby achieving remote sensing evaluation of rice disasters and yields [44]. On the other hand, the presence of turbid water or luxuriant algal growth on the water surface hinders the light transmission through the floodwater, which results in a decrease in photosynthesis [45]. However, few studies have focused on the photoenergy utilization efficiency of rice under flooding conditions, and the underlying physiological mechanisms need to be further explored.

### 3.5. Effects of Flooding Stress on Endogenous Hormones Balance

**Figure 4:**
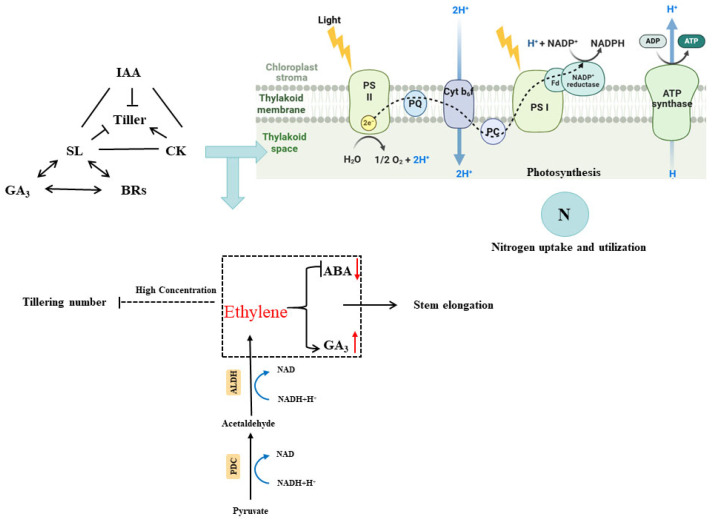
Effects of flooding stress on endogenous phytohormones in rice. Flooding enhances ethylene biosynthesis and transport. IAA and SL suppress tiller formation, whereas CK promotes it. The signaling pathways of SL, IAA, and GA_3_ interact to collectively modulate photosynthesis and nitrogen uptake and utilization. Ethylene reduces ABA content while increasing GA_3_ levels. This hormonal shift promotes internode elongation but inhibits tillering. Abbreviations: ABA: abscisic acid, GA: gibberellin, IAA: Indole-3-acetic acid, CK: cytokinin, SL: strigolactone.

In response to flooding, the accelerated elongation of rice stems is regulated not only by energy metabolism but also by the levels of endogenous hormones. It demonstrates that under flooding conditions, ethylene synthesis and transport in rice are enhanced, leading to an increase in ethylene concentration, which causes a decrease in abscisic acid (ABA) levels and an increase in gibberellin (GA_3_) levels, thereby promoting stem elongation (Figure 4) [46]. The imbalance between elevated ethylene levels and endogenous hormones influences tillering in plants (Figure 4) [47]. Previous research showed that the ethylene content in different genotypes of rice under semi-flooded waterlogging significantly increased, with an increase of 75% to 345%, and there were large differences between varieties. Our latest study showed that the growth trend of ethylene in different rice genotypes under flooding conditions was manifested as indica hybrid rice > conventional japonica rice > conventional indica rice ≥ Sub1 genotype > japonica hybrid rice. Under flooding conditions, the ethylene synthesis and transport in the rice body are both enhanced. The increase in the ethylene concentration not only regulates the occurrence of tillering, but also induces the formation of aeration tissue, stimulating the formation of roots and the hyperplasia of keratinous pores (Figure 4). These characteristics are all adaptation strategies of rice to flooded environments [48].

In addition, studies have shown that auxin (IAA) and cytokinin (CK) are involved in the regulation of rice tillering. IAA inhibits the occurrence of tillering, while CK promotes tillering growth (Figure 4) [49]. In addition, the newly discovered plant hormone strigolactone (SL) also has an inhibitory effect on the formation of rice tillering (Figure 4) [50]. At the physiological level, SL coordinates IAA and CK to regulate the occurrence of rice tillering (Figure 4). Regarding its mechanism of action, some scholars have proposed models based on auxin transport channels and the second messenger theory [51]; recent studies have shown that rice tillering mainly connects with IAA and CK, SL may also interact with the signal pathways of GA_3_ and rapeseed lactone (Figure 4) [52]. In addition, changes in endogenous hormones will also affect crop photosynthesis as well as nitrogen absorption and utilization (Figure 4) [53]. The effects of endogenous hormones on tillering described above are based on research conducted under normal water conditions, while under stress conditions, three hormones such as ethylene, abscisic acid (ABA), and gibberellic acid (GA_3_) play key roles in regulating tillering.

### 3.6. Effect of Flooding Stress on Nitrogen Utilization Efficiency

**Figure 5:**
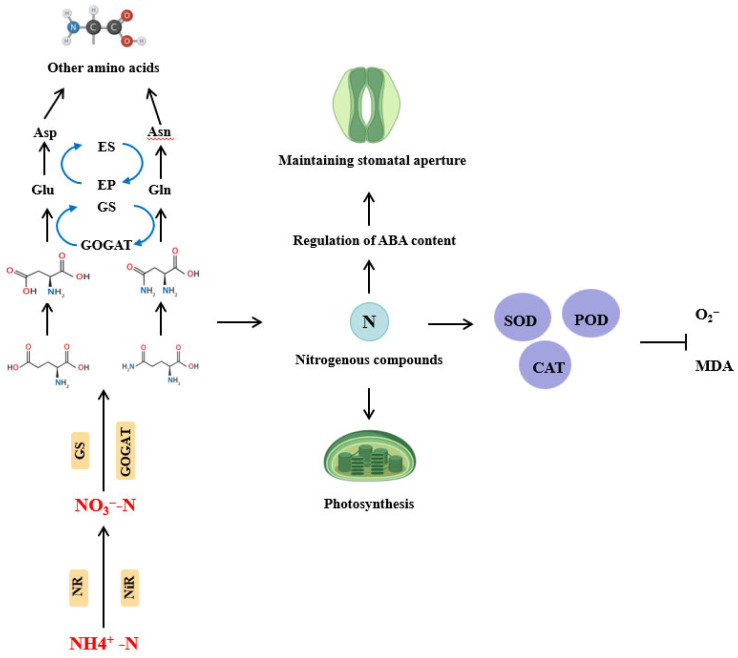
Impact of flooding stress on NUE in rice. Under flooding stress, soil nitrogen predominantly exists as NH_4_^+^-N. NO_3_^−^-N is reduced to ammonium via NR and NiR. Ammonium is assimilated into Glu and Gln through the GS/GOGAT cycle, with subsequent synthesis of Asp and Asn. Nitrogen assimilation products regulate stomatal aperture via modulation of ABA levels and mitigate oxidative stress by enhancing SOD, POD, and CAT activities, thereby reducing ROS and MDA accumulation. Furthermore, optimized nitrogen metabolism promotes photosynthetic efficiency under non-stress conditions. Abbreviations: NUE: nitrogen use efficiency, NR: nitrate reductase, NiR: nitrite reductase, Glu: glutamate, Gln: glutamine, Asp: aspartate, Asn: asparagine, GOGAT: glutamate, GS: glutamine synthase.

Nitrogen is the most important nutrient element that improves rice growth, quality, and yield [54]. The effects of nitrogen on crop photosynthesis, endogenous hormones, and reactive oxygen metabolism are mainly from the following pathways: (1) nitrogen is an important component of chlorophyll and directly influences the activity of various enzymes involved in both light and dark reactions of photosynthesis (Figure 5) [55]; (2) nitrogen maintains stomata opening by regulating the content of ABA and improves photosynthetic efficiency (Figure 5) [56]; and (3) nitrogen can increase enzyme activity such as SOD, POD, and CAT, accelerate the removal of reactive oxygen species, and reduce the MDA content and delay leaf aging (Figure 5) [57,58].

Under flooding conditions, the nitrogen absorption capacity of rice roots declines, affecting the activity of nitrogen metabolism-related enzymes and leading to reduced nitrogen uptake and utilization efficiency [59]. At the same time, the nitrogen transport capacity in rice is significantly reduced, and both the distribution and translocation of nitrogen within the plant are inhibited, leading to an imbalance in nitrogen allocation among different organs [60]. The absorption and utilization of nitrogen in rice are mainly inorganic nitrogen, including NO_3_^−^-N and NH_4_^+^-N, where NO_3_^−^-N is the main nitrogen source for crop absorption and utilization, while in soils with severe waterlogging or reducing properties, NH_4_^+^-N is the main nitrogen source [61]. The absorption and utilization of nitrogen by crops were completed through a series of nitrogen metabolic enzymes involved in the reaction and transformation. It was reported that nitrate reductase NR is the first enzyme and rate-limiting enzyme in the process of nitrate nitrogen reduction and assimilation (Figure 5). Nitrate (NO_3_^−^-N) absorbed by crops is reduced to ammonium (NH_4_^+^) through the actions of nitrate reductase (NR) and nitrite reductase (NiR). Then, under the action of glutamine synthase GS and glutamate synthase GOGAT, it is assimilated into glutamate and glutamine through the glutamate synthase cycle, further forming aspartic acid and asparagine, and finally forming other amino acids or nitrogen-containing compounds (Figure 5). The GS-GOGAT cycle is the main pathway for NH_4_^+^ assimilation in plants and is the center of the entire nitrogen metabolism. Finally, endopeptidase (EP) and exopeptidase act synergistically to degrade proteins, and changes in EP activity are closely associated with the process of protein degradation [62]. Therefore, many scholars have used these key enzymes NR, NiR, GS, GOGAT, and EP in the nitrogen absorption and utilization process as indicators for evaluating the rice yield and physiological mechanisms of nitrogen utilization efficiency [63,64]. At present, there are many studies focused on nitrogen absorption and accumulation in rice under flood stress, whereas reports on nitrogen use efficiency and its underlying physiological mechanisms remain limited.

### 3.7. The Impact of Flooding Stress on Rice Grain Yield

The rice yield reduction caused by flooding can reach 10–100%, depending on the flood intensity, depth, flood cycle, water temperature, water turbidity, light intensity in the water, soil fertility, the fertility stage of the crop during flooding, etc. [65]. Previous studies have shown that the severe yield reduction in rice during the tillering period is mainly due to the higher decrease in tillering in a single plant and the decrease in the number of ears. This decline in the number of ears is positively correlated with the depth of flooding, and is particularly prominent among varieties that are not water-resistant [66]. During the jointing and grain-filling periods, both the 1000-grain weight and the grain filling percentage of rice were significantly reduced [67]. Previous research showed that rice yield loss under semi-flooding is not only due to reduced tillering and the panicles number, but also declines in the LAI, growth rate, biomass, spikelets per panicle, and grain filling percentage. However, the relevant physiological mechanisms of the reduction in rice tillering, the panicles number, grain filling percentage, and grain yield under flood stress need to be further studied in depth.

## 4. Rice Stress-Resistant Breeding and Cultivation Regulation Methods

### 4.1. Breeding Strategies to Reduce the Stress of Rice Flooding

Significant progress was made in the identification of major quantitative trait locus (QTL) in rice chromosomes, which is the primary contributor for tolerance. Several QTLs, such as SUB1, AG1, AG2, SNORKEL1 (SK1), SNORKEL2 (SK2), etc., were identified in different chromosomes available for molecular marker technology to develop flood-tolerant varieties through a marker-assisted backcrossing (MABC) strategy [68]. The *Sub1* (Submergence-1) gene was identified from the flood-resistant Indian rice variety FR13A. The quantitative trait locus (QTL), located on rice chromosome 9, accounts for up to 70% of the difference between flood-resistant and flood-sensitive varieties [69]. Rice varieties with the *Sub1* gene imported (such as Swarna-Sub1, Sambha Mahsuri-Sub1, CR1009-Sub1, etc.) exhibit significantly improved flood tolerance [70]. Varieties introduced with the Sub1 gene can tolerate complete submergence for up to two 2 weeks during the vegetative growth period, because *Sub1* is regulated by ethylene levels [71], which can inhibit the production of ethylene during rice flooding, reduce the content of gibberellin, inhibit the rapid elongation between rice nodes in the flooded environment, and reduce the consumption of carbonated water [72]. Additionally, this gene enhances the ascorbic acid–glutathione cycle and accelerates the scavenging of reactive oxygen species and free radicals, thereby significantly improving rice survival under flooding conditions [73]. However, studies have shown that these modified varieties do not adapt well to semi-flooded conditions due to their short stature and slow growth under such environments [74,75].

Later, scientists discovered that the *AG* (Anaerobic germination) gene significantly improved the germination and seedling emergence rates of rice seeds under flooding conditions [76]. The *AG* genes include *AG1* and *AG2*, which activate α-amylase under anaerobic conditions, accelerating starch degradation into glucose. This process generates ATP via the ethanol fermentation pathway, thereby promoting seed germination [55].

SNORKEL1/SNORKEL2 (SK1/2) genes that initiate deepwater response by encoding ERFs involved in ethylene signaling were identified by Hattori et al. [77]. SK1/2 are absent in other cultivated rice and are found only in deepwater rice varieties [78]. The SK1/2-regulated downstream factors are yet to be known. SUB1 locus also consists of a cluster of three OsERFVIIs that are related to SK1/2 but works differently [79]. ERFVIIs SNORKEL1 and 2 transcriptionally regulate the escape strategy in certain deepwater cultivars while in other varieties, transcription factor OsEIL1 regulates gibberellin production [80].

Currently, flood-resistant varieties introduced with the *Sub1* gene improved, such as IR64-Sub1, Swarna-Sub1, BR11-Sub1, etc., have been widely promoted and planted in Southeast Asian and South Asian countries such as the Philippines, Bangladesh, and India. In recent years, many countries (including China) have introduced *Sub1* genotype rice for research, with most studies focusing on the physiological and molecular mechanisms of its flood tolerance [81]. However, its promotion and application remain limited in flood-prone regions such as the Yangtze River Basin. This is mainly due to the limited development and utilization of *Sub1* and *AG* genotype rice germplasm, highlighting the need to intensify efforts in using these genes to develop flood-tolerant, high-yield, and high-quality rice varieties [82].

In recent years, molecular marker-assisted selection (MAS) technology has been widely used in rice water-resistant breeding rice. MAS enables the pyramiding of waterlogging resistance genes and QTLs, allowing more efficient screening and utilization of superior germplasm and the development of new rice varieties with enhanced waterlogging tolerance [83]. Specifically, by developing molecular markers closely linked to waterlogging resistance genes, MAS technology enables the rapid screening of plants carrying these genes during breeding [84]. This method combines conventional breeding and molecular biology techniques, significantly improving breeding efficiency and shortening breeding cycles [85]. In addition, the rapid development of high-throughput genotyping and genome sequencing technologies is expanding the application of MAS technology while significantly reducing its costs [86]. In the future, MAS technology is expected to play an indispensable role in breeding water-resistant rice, providing stronger support for improving rice flood capacity and ensuring food security [87].

### 4.2. Cultivation and Regulation Methods to Alleviate Flooding Stress in Rice

#### 4.2.1. Drainage and Disaster Reduction

After a flooding threat, the primary measure is the timely drainage of excess water. There are two approaches to post-flood water management: one is to drain the excess accumulated water, and the other is to reduce the water level to the normal irrigation layer. The effects of these two methods on the recovery and growth of rice after flooding stress are quite different. In actual production, farmers usually drain all the accumulated water after the flooding, which is not conducive to the post-disaster recovery and growth of rice [88]. Once the water level returns to the normal layer, the root system gradually obtains access to oxygen, helping to alleviate oxidative stress damage.

#### 4.2.2. Optimize Fertilizer Management

Optimizing fertilizer management practices is another effective way to improve rice flood resistance. Previous research has shown that rice exhibits significant differences in flood resistance under various fertilizer management regimes. Excessive application of nitrogen fertilizer reduces the flood tolerance of rice seedlings [89]. When the soil phosphorus is deficient, increasing phosphorus fertilizer input can enhance the flood tolerance of rice [90]. Previous research showed that the best nitrogen fertilizer management can improve the stress resistance of rice under water flood conditions [91]. In practical production, fertilizer management is typically implemented after flood stress to promote the recovery and regeneration of rice seedlings [92]. However, less attention has been given to fertilizer management before and during flooding as a means to mitigate flood stress in rice.

#### 4.2.3. Exogenous Growth Regulatory Substances

Using exogenous growth regulatory substances to regulate crop growth is another effective approach to enhance crop stress resistance [93]. Seed priming and foliar spraying are two commonly used methods to alleviate the adverse effects of stresses on crops. Seed priming is an effective cultivation technique that rapidly promotes seed germination and establishment, enhances crop stress resistance, and increases the yield [94]. (1) Seed initiation involves soaking seeds in growth regulators to slow water uptake, keeping them swollen while activating metabolic processes and DNA repair, preparing them for germination without radicle emergence [95]. Seed-induced technology has been studied in improving the stress resistance of crops such as wheat, rice, corn, and sorghum [96]. (2) Leaf spraying involves applying appropriate concentrations of exogenous substances directly onto the crop’s foliage at specific growth stages to regulate growth. Previous studies have shown that nutrient elements, plant growth regulators, either as seed treatments or foliar sprays, can significantly enhance seed vigor, improve seedling establishment, regulate crop growth, and alleviate stress caused by adverse conditions [97]. Different studies mentioned that spraying exogenous hormones such as γ-aminobutyric acid and gibberellin can significantly alleviate the damage of waterlogging to rice plants and accelerate post-disaster recovery and growth [98]. Previous research showed that spraying exogenous γ-aminobutyric acid, salicylic acid, and GA_3_ can significantly improve the waterlogging resistance of rice, enhance the recovery of rice plants during and after semi-waterfall stress, and increase the yield (data not published).

## 5. Conclusions and Prospective

**Figure 6:**
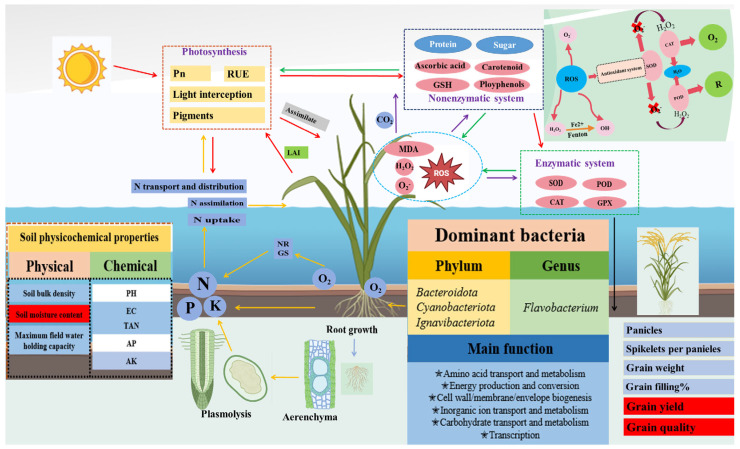
The multi-level linkage mechanism of rice under flooding cultivation across the “soil–root system–canopy–yield” continuum. Underground, root growth is modulated by soil physical and chemical properties (bulk density, pH, EC, TAN, AP, AK), forms aerenchyma to ease plasmolysis from waterlogging stress, and collaborates with Proteobacteria to boost nitrogen uptake and assimilation. In the canopy metabolic aspect, the photosystem captures light, optimizes pigments, and leverages a key enzyme network (marked by Pn/RUE) to convert CO_2_ to sugar and protein, while activating a redox balance system where enzymatic (SOD/POD/CAT/GPX scavenging O_2_^−^/H_2_O_2_) and non-enzymatic (ascorbic acid, Carotenoid, GSH, Polyphenols) pathways coordinate ROS homeostasis. In yield formation, nitrogen transport and distribution, mediated by phosphate and potassium ions, direct photosynthetic products to grain-related structures (spikelets number, spikelets per panicle, grain weight, grain filling percentage). The blue-based water areas and bidirectional arrows (O_2_/CO_2_ cycle, H_2_O_2_→H_2_O + O_2_ metabolic flow) clearly show how submerged environments achieve global coupling of energy and matter via microbial symbiosis, antioxidant defense, and carbon–nitrogen metabolic reprogramming.

Rice is a vital grain crop worldwide, but frequent flooding in southern rice-growing regions has caused significant yield losses. As mentioned in Figure 6, the flooding stress disrupts multiple interconnected factors including soil properties, microbial activity, root function, nutrient and water uptake, plant growth, radiation and nitrogen use efficiencies, oxidative stress balance, and reproductive development collectively resulting in substantial yield reductions in rice. It is worth noting that the formation of aerenchyma is one of the adaptation strategies for rice under flooding stress. Despite advances in understanding rice responses to flooding and developing resistant varieties, key challenges remain to improve flood tolerance and protect yields for food security.

Future research on rice flooding resistance should emphasize adopting new technologies, broadening research perspectives, developing novel theories, and enhancing interdisciplinary collaboration. Multi-omics, gene editing, and AI-based phenotyping technologies are used to identify and optimize flood-resistant genes and rapidly monitor rice growth and physiology under flooding stress. In addition, we expand our research perspective by studying the combined effects of flooding stress and other adverse stresses on rice yield and quality as well as developing effective cultivation management strategies. Simultaneously, a comprehensive evaluation system for rice flooding resistance was developed, a new flooding resistance hypothesis proposed, and the relationship between flooding resistance and ecological adaptability was analyzed. Moreover, we enhance interdisciplinary collaboration to study soil changes, ecosystem impacts, and flooding patterns under climate change, providing a scientific basis for improving rice flood-resistant breeding and cultivation. By applying new technologies, expanding research perspectives, proposing new theories, and strengthening interdisciplinary cooperation, we comprehensively improve rice flood resilience, enhance disaster forecasting, and support high yields.

## Data Availability

The original contributions presented in this study are included in the article. Further inquiries can be directed to the corresponding author.
